# A Unique Case of High-Grade Dedifferentiated Melanoma Without a Known Primary Site

**DOI:** 10.7759/cureus.66951

**Published:** 2024-08-15

**Authors:** Shawn Keating, Riddhi Machchhar, Ujjwala Jain, Jordan Lipschutz, Gabriela Naronowicz, Moiuz Chaudhri, Anish Kanukuntla

**Affiliations:** 1 Internal Medicine, Hackensack Meridian Ocean Medical Center, Brick, USA; 2 Internal Medicine, Ocean University Medical Center, Brick, USA

**Keywords:** alkaline phosphatase (alp), sarcoma, occult primary melanoma, dedifferentiated melanoma, malignant melanoma metastasis

## Abstract

Melanoma is a malignant neoplasm that arises in melanocytes, pigment-producing cells present primarily in the skin. However, not all malignant melanomas originate from the skin, and the other sites of origin include the uveal (eye) or mucosa (rectal or oral); it can have different patterns of mutations. While targeted therapies and immunotherapies have transformed the treatment of this disease in the metastatic setting, resistance mechanisms can still pose challenges for patients and their healthcare providers. We present a case of a male patient with metastatic melanoma and discuss its clinical presentation, diagnostic workup, treatment options, and outcomes. By exploring the complexities of this multifaceted disease process, clinicians and researchers can gain valuable insights into potential areas for improved management strategies. Ultimately, we should aim to deepen our understanding of melanoma and work towards better patient outcomes.

## Introduction

Oncology faces significant challenges in the management of metastatic melanoma due to its aggressive nature and tendency for widespread dissemination. Melanoma, which arises from the malignant transformation of melanocytes, accounts for a substantial proportion of skin cancer-related deaths worldwide. As per the American Cancer Society, melanoma accounts for 1% of skin cancers but causes the great majority of skin cancer-related deaths. The Centers for Disease Control and Prevention and the National Cancer Institute collectively list it as the fifth most common cancer type, accounting for 5% of new cancer diagnoses.

Despite vast improvements in the early detection and treatment of primary melanomas, metastatic melanoma remains challenging, with few treatment options and generally poor prognosis [1]. Recent advances in targeted therapies and immunotherapies have revolutionized the management of metastatic melanoma. However, despite these breakthroughs, many patients still experience disease progression due to intrinsic and acquired resistance mechanisms [2]. In this report, we discuss a case of a patient who presented with recurrent metastatic melanoma, in whom a primary lesion was never found in both instances. In the second bout of the disease, a rare histological appearance of high-grade dedifferentiated melanoma was observed. We believe our findings will add to the growing body of knowledge on metastatic melanoma.

## Case presentation

**Figure 1:**
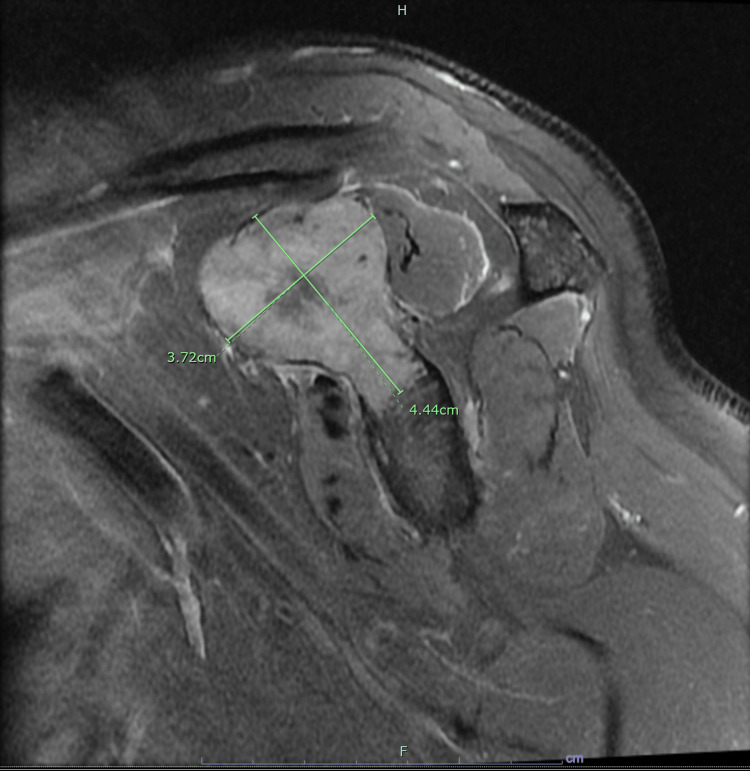
MRI without contrast of the left shoulder, left scapula Multiple metastatic masses are seen among the shoulder with the largest in the scapula at the level of the coracoid process measuring 3.7 x 4.4 cm MRI: magnetic resonance imaging

**Figure 2:**
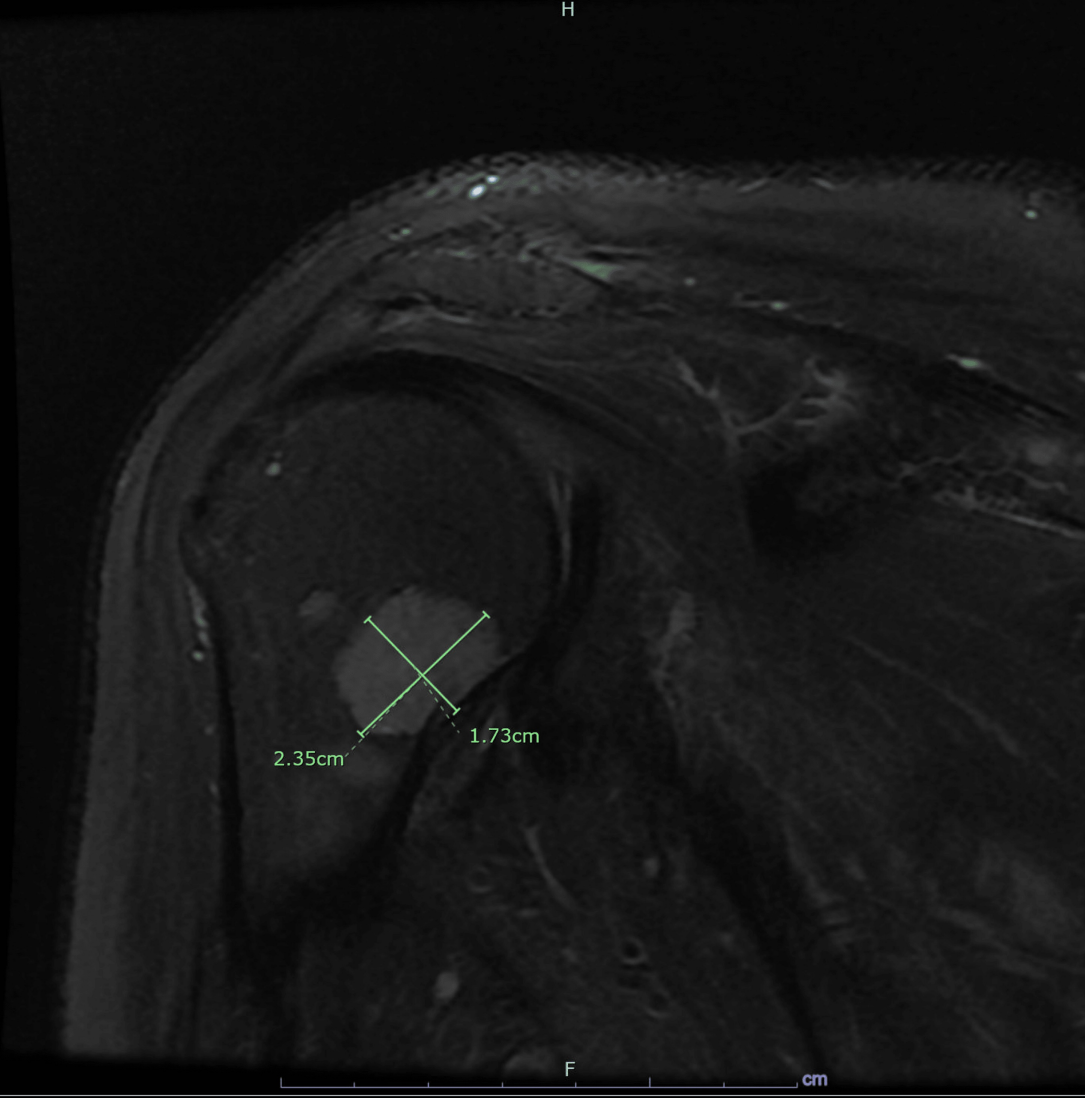
MRI without contrast of the left shoulder, left proximal humerus Additional smaller masses are seen within the proximal humerus with the largest measuring 1.7 x 2.4 cm MRI: magnetic resonance imaging

This patient was a 59-year-old male with a past medical history of stage IV melanoma with single brain metastasis 13 years ago, stage II esophageal adenoma status-post resection performed six years ago, hypertension (HTN), and gastroesophageal reflux disease (GERD) who initially presented with an abnormal elevation of alkaline phosphatase (2540 U/L, normal range: 38-126 U/L). His left shoulder had been causing discomfort for the past three months, which he associated with his household chores. Also, the patient reported feeling increasingly fatigued during this time. He had sought the advice of an orthopedic surgeon, who suggested that it might be connected to a previous rotator cuff injury. Despite undergoing physical therapy, the patient had not experienced any relief, and diagnostic imaging had not revealed any pathology. At this time, the oncologist advised the patient to go to the hospital. Bone scan and other imaging showed multiple lesions throughout the skeletal system, including a rather large lesion in the left scapula area (Figure 1) and additional smaller masses in the left proximal humerus (Figure 2). After taking a biopsy of this large bone lesion, the patient returned home to await the biopsy results.

The biopsy results yielded histologic sections showing a biphasic tumor comprising a relatively low-grade hyaline cartilage component juxtaposed to a high-grade epithelioid and anaplastic neoplasm with round nuclei and prominent nucleoli supported by rich stromal vasculature. Immunohistochemistry performed was negative for Melan-A, HMB45, MITF, and BRAFV600E. H3K27me3, seen in 37% of melanomas [3], showed aberrant loss of nuclear expression. The tumor was also negative for S100, SOX10, anti-cytokeratin cocktail (AE1 and AE3), CAM5.2, desmin, and CD34. The tissue sample pattern was that of high-grade dedifferentiated melanoma. The patient returned to the hospital a few weeks later due to a worsening overall condition. Eventually, he opted for home hospice rather than continue with possible treatment options due to his declining health; he died six days later.

## Discussion

We presented the unique and challenging case of a patient diagnosed with a poorly differentiated/dedifferentiated malignant neoplasm, clinically thought to represent a recurrent/metastatic dedifferentiated melanoma. High-grade dedifferentiated melanoma is known for its aggressive behavior and poor prognosis. Dedifferentiated melanomas are biphasic tumors that show a transition between conventional melanoma components and undifferentiated areas with histopathological and immunohistochemical features of other cell lineages. The dedifferentiated portion of these melanomas will appear as atypical fibroxanthoma/undifferentiated pleomorphic sarcoma. These abnormal tumors have also been reported to possess features seen in carcinomas, leiomyosarcoma, rhabdomyosarcoma, ganglioneuroblastic tumors, other sarcomas, and spindle cell neoplasms [4]. The histologically unusual presentation led to a delay in identifying the specific type of malignancy as the samples had to be sent to a tertiary center. Reports have shown the rarity of high-grade dedifferentiated melanoma [5].

Occult primary melanoma is relatively rare, accounting for only a small proportion of melanoma cases: approximately 3.2% of cases in the United States per year [6]. In our patient, despite extensive clinical examination and investigations, including dermatoscopy, imaging studies, and histopathological analysis, the primary lesion remained elusive. The most popular theory that explains the inability to find the primary tumor is that it regresses into the body, most commonly seen in white men aged 40-59 years [7], the demographic our patient fell into.

New drugs and drug combinations are being developed to improve the treatment of metastatic melanoma, as researchers have found secondary resistance to existing treatments like tyrosine kinase inhibitors (RTKs), especially those directed at activating BRAF mutations, CTLA4, and PD1 inhibitors. There is ongoing research aimed at pinpointing biomarkers that can anticipate individual patient responses to treatment. This approach would enable the development of tailored and optimal treatment plans based on unique mutational and biomarker profiles [8]. Personalized treatment can improve patient prognosis and reduce the cost of treatment by administering only those drugs that are likely to have an effect, ultimately reducing the suffering of patients from trial therapies [8-9].

The challenging nature of high-grade dedifferentiated melanoma, combined with occult primary melanoma, underscores the importance of a multidisciplinary team involving dermatologists, oncologists, pathologists, and radiologists [8-10]. Also, when examining patients with melanoma of unknown origin, it is important to revisit and explore previous excision materials and conduct various tests, including endoscopic exams of the nose, larynx, and gastrointestinal system, physical exams of the anus and genital area, and CT scans of the brain, neck, thorax, and abdomen. For female patients, gynecological exams are recommended, and a Wood lamp examination may be necessary in some cases. Consulting with different departments, such as gastroenterology, ophthalmology, dermatology, and ENT, may also be required [9-10]. However, it is important to note that even with all of these tests and consultations, the origin of melanoma may remain unknown [10]. Clinical trials might have been an option if our patient had been diagnosed earlier before so much extensive disease and deconditioning. Furthermore, an interdisciplinary conversation about improved morbidity and mortality may have been possible.

## Conclusions

High-grade dedifferentiated melanoma is a type of melanoma with a poor prognosis due to its aggressive behavior. It is a biphasic tumor that shows a transition between conventional melanoma components and undifferentiated areas. The dedifferentiated portion of these melanomas can resemble various other types of tumors. To create an accurate report on high-grade dedifferentiated melanoma, gathering all relevant data, analyzing it, and properly citing all sources is important. Furthermore, as primary tumors have unique presentations and elusive histopathology, clinicians need to be diagnostically efficient and clinically contemplate various diagnoses in light of a nondescript presentation of fatigue and musculoskeletal dysfunction.
